# Advances in phytochemical and modern pharmacological research of *Rhizoma Corydalis*

**DOI:** 10.1080/13880209.2020.1741651

**Published:** 2020-03-29

**Authors:** Bing Tian, Ming Tian, Shu-Ming Huang

**Affiliations:** a Department of Neuroscience, Institute for Chinese Medicine, Heilongjiang University of Chinese Medicine, Harbin, China; b Experimental Training Center, Heilongjiang University of Chinese Medicine, Harbin, China

**Keywords:** Traditional Chinese medicine, bioactive components, phytochemistry, pharmacological effects

## Abstract

**Context:**

*Rhizoma Corydalis* (RC) is the dried tubers of *Corydalis yanhusuo* (Y. H. Chou and Chun C. Hsu) W. T. Wang ex Z. Y. Su and C. Y. Wu (Papaveraceae). Traditionally, RC is used to alleviate pain such as headache, abdominal pain, and epigastric pain. Modern medicine shows that it has analgesic, anti-arrhythmia, and other effects.

**Objective:**

We provided an overview of the phytochemical and pharmacological properties of RC as a foundation for its clinical application and further research and development of new drugs.

**Methods:**

We collected data of various phytochemical and pharmacological effects of RC from 1982 to 2019. To correlate with existing scientific evidence, we used Google Scholar and the journal databases Scopus, PubMed, and CNKI. ‘*Rhizoma Corydalis*’, ‘phytochemistry’, and ‘pharmacological effects’ were used as key words.

**Results:**

Currently, more than 100 chemical components have been isolated and identified from RC, among which alkaloid is the pimary active component of RC. Based on prior research, RC has antinociceptive, sedative, anti-epileptic, antidepressive and anti-anxiety, acetylcholinesterase inhibitory effect, drug abstinence, anti-arrhythmic, antimyocardial infarction, dilated coronary artery, cerebral ischaemia reperfusion (I/R) injury protection, antihypertensive, antithrombotic, antigastrointestinal ulcer, liver protection, antimicrobial, anti-inflammation, antiviral, and anticancer effects.

**Conclusions:**

RC is reported to be effective in treating a variety of diseases. Current pharmacological studies on RC mainly focus on the nervous, circulatory, digestive, and endocrine systems, as well as drug withdrawal. Although experimental data support the beneficial effects of this drug, its physiological activity remains a concern. Nonetheless, this review provides a foundation for future research.

## Introduction

*Rhizoma Corydalis* (RC), also known as *Corydalis yanhusuo*, *YuanHu*, *YanHu*, or *XuanHu* in China, is a well-known traditional Chinese medicine (TCM) prepared from the dried tubers of *Corydalis yanhusuo* (Y. H. Chou and Chun C. Hsu) W. T. Wang ex Z. Y. Su and C. Y. Wu (Papaveraceae). (Wang et al. 2016). RC has a long history of medicinal use and is mainly cultivated in the Zhejiang, Jiangxi, and Anhui provinces of China. RC was first recorded in Shennong Herbal Classic and was listed as a medium-grade drug. RC is pungent, bitter, and warm, and is transported to the spleen and the liver meridians. In TCM, RC is believed to have functions such as activating blood, reinforcing vital energy, and relieving pain (Chinese Pharmacopoeia Commission (CPC) 2015). In the clinical practice of Chinese medicine, RC often appears as a compound. Alkaloids are important biological active constituents of RC (Wu et al. 2012), including tertiary amines, quaternary alkaloids, and many non-alkaloids. Presently, more than 80 alkaloids have been isolated and identified from RC (Xiao et al. 2011; Zhou et al. 2012). Modern medical research has revealed that RC has significant analgesic, sedative, and hypnotic effects, and in a variety of diseases such as arrhythmia, gastric ulcer, and coronary heart disease, it displays a good clinical effect (CPC 2015; Wang et al. 2016). To date, however, there has been no comprehensive review on the phytochemical and the pharmacological effects of RC. Based on the high therapeutic value of RC, we sought to systematically summarize the latest findings regarding the phytochemical and pharmacological effects of RC and its bioactive components between 1982 and 2019 by using Google Scholar and the journal databases Scopus, PubMed, and CNKI, in an attempt to provide a foundational knowledge guide for its subsequent research and utilization.

## Phytochemistry

To date, more than 100 compounds have been isolated and identified from RC (Wu et al. 2012). Alkaloids are important biological active constituents of RC, including tertiary amines and quaternary alkaloids. Besides alkaloids, there are additionally many non-alkaloid components, including organic acids, steroids, carbohydrates, and other chemical compounds (Liu et al. 2013). To better understand the physiological effects of different active ingredients of RC, the primary components of the compound are shown in Table 1.

**Table 1.** The main chemical constituents isolated from RC.

Category	Number	Ingredient name	Reference
Protoberberine alkaloids	**1**	Dehydrocorydaline	(Tong et al. 2005)
	**2**	Palmatine	
	**3**	Coptisine	
	**4**	Columbamine	
	**5**	Corydayanine	(Zhou et al. 2012)
	**6**	Yanhusuine	
	**7**	Corydaline	(Xiao et al. 2011)
	**8**	D-corydaline	
	**9**	Berberine	
	**10**	Jatrorrhizine	
	**11**	Tetrahydroberberine	(Cui et al. 2018)
	**12**	Berberrubine	
	**13**	Tetrahydrocolumbamine	(Du et al. 2018)
	**14**	Epiberberine	
	**15**	Scoulerine	(Mi et al. 2016)
	**16**	Isocorypalmine	(Ma et al. 2008)
	**17**	13-Methylpalmatine	
	**18**	Dehydrocorybulbine	(Wei et al. 2016)
	**19**	Demethylcorydalmine	
	**20**	13-Methylpalmatrubine	
	**21**	D,L-Tetrahydropalmatine	
	**22**	D,L-Tetrahydrocoptisine	
	**23**	13-Methoxyberberine	(Zheng et al. 2018)
	**24**	*N*-Methylcanadine	
	**25**	Yuanhunine	
	**26**	Corybulbine	
	**27**	Tetrahydrocolumbamine	
	**28**	Corydalmine	
	**29**	Scoulerine	
	**30**	Isocorybulbine	
	**31**	Isocorypalmine	(Ma et al. 2008)
	**32**	Corypalmine	
	**33**	8-Oxocoptisine	(Hu et al. 2009)
	**34**	Stepharanine	
	**35**	Tetrahydrojatrorrhizine	(Sun et al. 2014)
	**36**	Demethyleneberberine	
	**37**	Worenine	
	**38**	Tetrahydrocorysamine	(Zhou et al. 2016)
	**39**	Dehydroyanhunine	(Wu et al. 2015)
	**40**	8-Trichloromethyl-7,8-dihydrocoptisine	
	**41**	Dehydrocorybuldine	
Aporphine alkaloids	**42**	Glaucine	(Lei et al. 2013)
	**43**	Oxoglaucine	(Du et al. 2018)
	**44**	D-glaucine	(Wei et al. 2016)
	**45**	Corunine	(Cheng et al. 2008)
	**46**	7-Formyldidehydroglaucine	(Hu et al. 2009)
	**47**	Didehydroglaucine	
	**48**	Nantenine	
	**49**	*O*-Methylbulbocapnine	
	**50**	Dehydroglaucine	(Zhou 2012)
	**51**	Pontevedrine	
	**52**	*N*-methyllaurotetanine	
	**53**	Pulbocapnine	(Feng et al. 2018)
	**54**	Norglaucine	
	**55**	Oxoglaucidaline	
	**56**	Isoboldine	(Wu et al. 2015)
	**57**	Dehydronantenine	
	**58**	Thaliporphine	
	**59**	Lirioferine	
	**60**	Pontevedrine	
Opiates alkaloids	**61**	Protopine	(Xiao et al. 2011)
	**62**	Cryptopine	(Zheng et al. 2018)
	**63**	α-Allocryptopine	(Zhang et al. 2015)
	**64**	Nordelporphine	(Zhou 2012)
Other alkaloids	**65**	Tetrahydroprotopapaverine	(Wei et al. 2016)
	**66**	Fumaricine	
	**67**	Chelerythrine	(Zheng et al. 2018)
	**68**	Dihydrosanguinarine	
	**69**	Dihydrochelerythrine	
	**70**	Noroxyhydrastine	(Hu et al. 2009)
	**71**	Leonticine	(Zhou 2012)
	**72**	Taxilamine	
	**73**	6-Acetonyl-5,6-dihydrosanguinarine	(Zhang 2008)
	**74**	Homochelidonine	(Wu et al. 2015)
	**75**	Saulatine	
	**76**	Bicuculline	
	**77**	*N*,*N*-dimethyl-*N*′-methyl-diphenyl-one	(Feng et al. 2018)
	**78**	*N*,*N*-dimethyl-*N*′,*N*′,-dimethyl-diphenyl-one	
	**79**	*N*-methyl-tetrahydropalmatine	
	**80**	*N*-methyl-canadine	
	**81**	Coryphcnanthrinc	
Steroid	**82**	β-Sitosterol-3-*O*-β-D-glucopyranoside	(Zhou 2012)
	**83**	Stigmasterol	(Shi et al. 2011)
	**84**	β-Sitosterol	
	**85**	Daucosterol	
	**86**	Ergosta-4-en-3-one	(Zhang 2008)
Oganic acid	**87**	Succinic acid	(Liu et al. 2013)
	**88**	2-Hydroxy propionic acid	
	**89**	Palmitic acid	
	**90**	Octadecanoic acid	
	**91**	Citric acid	
	**92**	2,3-Dihydroxypropionic acid	
	**93**	Malic acid	
	**94**	Behenic acid	(Zhou 2012)
	**95**	Vanillic acid	(Zhang 2008)
	**96**	*P*-Hydroxybenzoic acid	
Carbohydrates	**97**	Polysaccharide YhPS-1	(Tao and Tian 2006)
	**98**	D-Lactose	(Liu et al. 2013)
	**99**	α-D-Glucopyranose	
	**100**	β-D-Glucopyranose	
Others	**101**	Pseudodehydrocorydaline	(Zheng et al. 2018)
	**102**	Phosphoric acid	(Liu et al. 2013)
	**103**	Ribonic acid-1,4-lactone	
	**104**	3,4-Dihydroxypropionic,2-carbonyluran	
	**105**	Glycerin	
	**106**	Cyclohexanehexol	
	**107**	Adenosine	(Hu et al. 2009)
	**108**	*N*(5)-Acetylornithine	
	**109**	Xanthosine	(Zhou 2012)
	**110**	Phenylalanine	
	**111**	Emodin	(Shi et al. 2011)
	**112**	Physcion	
	**113**	3β-Hydroxy-olean-11,13(18)-dien-2	

### Alkaloids

Alkaloids are the major biologically active components of RC. Presently, more than 80 alkaloids have been isolated and identified from RC (Xiao et al. 2011; Zhou et al. 2012); with benzylisoquinoline alkaloids account for a large proportion, including protoberberines, aporphines, opiates among others. Protoberberine alkaloids are the most abundant benzylisoquinoline alkaloids in RC (Zhou et al. 2012).

#### Protoberberines

The protoberberine alkaloids are composed of the tetracyclic rings system based on dibenzoquinolizine system, which is the ‘berberine bridge’ carbon formed by benzylisoquinolines coupled with the isoquinoline *N*-methyl group after phenol oxidation (Wang et al. 2019). To date, 41 protoberberine compounds have been found in RC. It contains tertiary amine bases such as tetrahydropalmatine (**21**) and quaternary ammonium bases such as berberine (**9**). Among them, tetrahydroberberine (THB), corydaline, tetrahydropalmatine (THP), and berberine (Ber) which have a significant analgesic effect (Wang et al. 2013; Guo et al. 2014), and dehydrocorydaline (DHC), tetrahydrocoptisine (THC) and coptisine having an anti-coronary heart disease and anti-inflammatory properties (Ishiguro et al. 2011; Li et al. 2013).

#### Aporphine alkaloids

Aporphine alkaloids are the basic parent nucleus of isoquinoline, which is a tetracyclic aromatic basic skeleton, formed by the phenolic oxidation coupling of phenyl isoquinoline precursors, and contain tertiary amine bases such as glaucine (**42**). So far, 19 compounds have been isolated from RC and identified. Modern pharmacological studies have shown that aporphine alkaloids have a variety of biological activities including anticancer, antiviral, antimicrobial, and anti-inflammatory among others (Ge and Wang 2018).

#### Opiates alkaloids

Opiates alkaloids in RC are attached to -OMe or -OCH2O-ring structures at C-10 and C-11 sites of the parent nucleus of proptopine (**61**) (Feng et al. 2018). At present, four compounds have been isolated and identified, among which protopine has been reported to have significant antihepatotoxic, anti-malaria, and acetylcholine inhibitory effects (Wangchuk, Keller, Pyne, Sastraruji, et al. 2012; Wangchuk, Keller, Pyne, Willis, et al. 2012; Still et al. 2013).

#### Other alkaloids

In addition to the abovementioned alkaloids, there are a few other isoquinoline alkaloids in RC, such as dihydrosanguinarine (**68**) saulatine (**75**), bicuculline (**76**), and other alkaloids.

### Non-alkaloids

As per previous studies, in addition to a large number of alkaloids, RC contains an abundance of steroids, carbohydrates, organic acids, phlegmatic temperament, amino acids, volatile oils, and trace elements (Liu et al. 2013). At present, 32 compounds have been identified.

## Pharmacological action

### Effects on the Central nervous system

#### Antinociceptive activity

Pain is an unpleasant emotional experience and feeling associated with tissue injury or potential tissue injury (Woolf 2010). Because existing analgesics such as potent opiate drugs cause severe side effects (Crofford 2010), for centuries, many different extracts from natural medicines, mainly plants, have been used for pain relief. RC is officially listed in the Chinese Pharmacopoeia (CHP) and its decoction and powder have been widely used to treat spastic pain, abdominal pain, and pain due to injury (CPC 2015). To add, it is referred to as ‘morphine’ in Chinese herbal medicine. Several studies from China showed that RC can act on γ‐aminobutyric acid (GABA) or opioid receptors, thereby alleviating painful symptoms (Li et al. 2012).

Some alkaloids from RC were recently discovered to have a profound effect on the dopaminergic system within the central nervous system, thereby playing an important role in the regulation of nociception (Ma et al. 2008). Dopamine transmission has been reported to play a central role in modulating pain perception in the supraspinal and spinal regions (Wood 2008). Wang et al. (2010) used a formalin-induced model to evaluate the analgesic mechanism of *l*-THP, the levo isomer of THP. They concluded that *l*-THP and its analogues can mediate the striatum/nucleus accumbens-arcuate nucleus-periaqueductal grey pathway through the blockade of dopamine D_2_ receptors to regulate nociception. Purified or synthesized *l-*THP has been approved and is used in China under the name, Rotundine, for a variety of clinical indications (CPC 2015). Wang et al. (2016) systematically evaluated the antinociceptive properties of RC by testing it in different pain modes, namely acute, inflammatory, and chronic pain, and explored the mechanism of RC activity. Their results indicated that RC effectively attenuated acute, inflammatory, and neuropathic pain, without causing tolerance. *In vitro*, RC exhibited prominent dopamine receptor antagonistic properties. In dopamine D_2_ receptor knockout mice, its antinociceptive effects were attenuated in acute and neuropathic pain but not in the inflammatory pain assays. It is noteworthy that RC additive is sold as a dietary supplement in the United States due to its low toxicity. Zhang et al. (2015) investigated the analgesic effects of *l*-THP in rats with bone cancer pain induced by tumour cell implantation (TCI). Studies have shown that high doses of *l*-THP (60 mg/kg) can significantly prevent or reverse bone cancer-related pain behaviours, and the activation of microglia cells caused by TCI and the increase in TNF-α and IL-1β levels can be inhibited by *l*-THP. However, *l*-THP failed to affect the activation of astrocytes and the increase in IL-1β induced by TCI. These data suggest that the analgesic effects of *l*-THP on bone cancer pain may underlie the inhibition of microglial cell activation and the increase in pro-inflammatory cytokine.

In a group of clinical control trials, Yuan et al. (2004) induced tonic pain using a cold-pressor, and then a single, oral administration of the extracts of RC (low dose: 3.25 g; high dose: 6.5 g). The results showed that pain intensity and pain scores significantly decreased (*p* < 0.01), which may have a clinical value in the treatment of mild to moderate pain. Qiu et al. (2009) compared the analgesic effect between RC processed with vinegar and cleansing RC through mice hot-board and acetic-acid-induced twisting experiments with a water decoction extract administered intragastrically. The results showed that the analgesic effect of RC using vinegar was significantly better than the cleansing RC. This indicated that the acetate formed by the combination of alkaloids and acetic acid in RC was easy to extract and dissolve.

#### Sedative and anti-epileptic activity

Most sedative drugs act on the brainstem network. Lu (2011) found that *l*-THP can significantly reduce spontaneous and passive activities in mice, block some descending sexual functions of brainstem reticular structure, inhibit conditioned reflex, prolong sleep time of cyclohexanobarbital sodium, resist the excitatory effect of small amounts of phenylpropylamine, and reduce the toxicity of large amounts of phenylpropylamine.

Epilepsy is a chronic disease where a sudden abnormal discharge of brain neurons leads to transient brain dysfunction. Chang and Lin (2001) examined the effect of *l*-THP on epilepsy and discussed the mechanism whereby it acts. Picrotoxin blocked the central inhibitory transmitter, GABA, and increased the release of dopamine in the amygdala. They found that injecting picrotoxin (3–4 mg/kg) could increase the exercise capacity of free activity and the height of turning in rats. Posture freeze was restrained and amygdaloidal release of dopamine was increased in anesthetized rats. After pre-treatment with *l*-THP, the picrotoxin-induced activity indexes were suppressed. To add, *l*-THP could inhibit seizure by inhibiting the release of dopamine in the amygdala.

In another study, Lin et al. (2002) performed a quantitative study of seizure activity in non-anesthetized rats with an ultrasound motion-sensing system. Their results indicate that *l*-THP can inhibit the release of dopamine from the amygdala at doses of 20–30 mg/kg, thereby preventing the epileptic attack by picrotoxin in the rat model. *l*-THP is thus an effective antiepileptic and could inhibit the development of amygdala kindling seizures in rats.

#### Anti-depression and anti-anxiety activity

Due to the pressures of life and work in modern society, the number of patients with depression continues to increase year by year. Hence, treatment for depression has become one of the research hotspots in the medical field. Sun et al. (2016) used chronic mild stress (CMS) for 14 days to establish a depression-like mouse model. They used the forced swim test (FST) to evaluate the efficacy of RC in depression, and used UPLC coupled with a triple-quadrupole mass spectrometer to assess plasma corticosterone levels. The proliferation rate and apoptosis of hippocampal precursor cells were detected by BrdU incorporation and terminal deoxynucleotidyl-transferase (TdT)-mediated dUTP nick end-labeling (TUNEL) staining. *In vitro*, the proliferation of neural stem cells (NSC) was assessed using the MTS assay. The results showed that RC inhibited the increase in plasma corticosterone levels, decrease in proliferation of hippocampal precursor cells, and body weight loss. RC also reversed hippocampal cell apoptosis and exhibited an antidepressant-like effect in CMS-induced mouse models of depression. RC could also promote neuroproliferation *in vitro*. These data indicate that RC can ameliorate depression. Its mechanism of action may include regulation of the hypothalamic–pituitary–adrenal (HPA) axis, reduction of stress hormone levels, promotion of hippocampal neuronal plasticity, and inhibition of apoptosis. Wu et al. (2015) used ^1^H-NMR metabolomics to study the antidepressant effect of total alkaloids (YHTA) in a chronic unpredictable mild stress (CUMS) rat model. Thirteen biomarkers in CUMS rats were identified through plasma metabolic profiling and multivariate data analysis. After YHTA treatment, the abnormal metabolites mentioned above in the model group and YHTA treatment group showed a tendency to return to normal levels. Such findings suggest that YHTA has a good therapeutic effect on depression.

Anxiety neurosis is a type of neurosis characterized by anxiety and physical symptoms such as palpitations, nervousness, fear, palpitations, and shortness of breath. Anxiety disorders are the most common mental disturbances. Leung et al. (2003) evaluated the anti-anxiety properties of orally-administered *l*-THP in a mouse model using the elevated plus-maze, hole board, and the horizontal-wire tests. The results showed that a low dose of THP (0.5–10 mg/kg) could significantly reduce anxiety in mice, avoid sedation myorelaxation, and produce anti-anxiety effect mediated by GABA_A_ receptor benzodiazepine site (BDS). Henkes et al. (2011) found that intraperitoneal injection of THP (25 mg/kg) could significantly reduce anxiety and motor movements. In addition, 3 mg/kg flumazenil did not completely antagonize the effects of THP.

#### Effect on acetylcholinesterase (AChE)

Acetylcholine (ACh) plays an important role as a neurotransmitter in the central nervous system. Xiao et al. (2011) isolated 8 isoquinoline alkaloids from the methanol extract of RC tubers using spectroscopic techniques. Thus, coptisine, Ber, palmatine, DHC, and jatrorrhizine were found to display AChE inhibitory effect, with half-maximal inhibitory concentration (IC_50_) values 1.01 ± 0.03, 0.47 ± 0.01, 0.74 ± 0.06, 0.62 ± 0.05, and 2.08 ± 0.09 µM, respectively.

According to the cholinergic system hypothesis, Alzheimer’s disease (AD) is a result of a decrease in acetylcholinergic levels in the cerebral cortex. Therefore, inhibiting AChE activity is a common treatment option for AD. Zhang et al. (2018) studied the neuroprotective mechanism of THP on ketamine-induced learning and memory impairment in mice. After treatment with THP, ketamine-induced AChE activity in mice was significantly reduced, while the decrease in ACh level was reversed. Such findings indicated that THP protected nerve cells in mice from apoptosis induced by ketamine. In addition, the Morris water maze (MWM) test and open field test were used to analyze learning and memory impairment in mice. Enzyme-linked immunosorbent assay (ELISA) kits and western blotting (WB) were employed to analyze oxidative stress, inducible nitric oxide synthase (iNOS), inflammation factors, glial fibrillary acidic protein (GFAP), caspase-3 and caspase-9, glial cell derived neurotrophic factor (GDNF), cytochrome c, and phospholipase C (PLC) γ1 protein expression. After treatment with THP, escape latency was significantly decreased in ketamine-induced mice. To add, the expression of oxidative stress, inflammation, and the above proteins could be inhibited. Through the mechanisms of anti-oxidation, anti-inflammation, and anti-apoptosis, learning and memory impairment induced by ketamine in mice can be ameliorated. Cao et al. (2018) demonstrated that *l*-THP can inhibit spatial memory impairment induced by low and high doses of methamphetamine in mice. In earlier studies, Qu et al. (2016) also demonstrated that THP could improve D-galactose-induced memory impairment in rats by inhibiting the activity of AChE. Wangchuk, Keller, Pyne, Sastraruji, et al. (2012) proved that isoquinoline alkaloids extracted and isolated from *Corydalis crispa*: protopine, also have AChE inhibitory activities.

#### Drug abstinence activity

Despite the substantial amount of work carried out in drug development, the treatment of cocaine and opioid addiction remain as unmet medical needs. To add, there are no cocaine withdrawal drugs approved by the FDA. Indeed, RC and its main effective components can improve the drug withdrawal syndrome of cocaine and opioid addiction.

*l*-Isocorypalmine (*l*-ICP) is a mono-demethylated analogue of *l*-THP extracted from RC; it, acts as a D_1_ partial agonist and a D_2_ antagonist *in vivo*. Xu et al. (2013) tested the effects of *l*-ICP on sensitisation, hyperactivity, and conditioned place preference (CPP) in cocaine-induced mice by receptor binding, cyclic adenosine monophosphate (cAMP), and GTPγS assays. The metabolites of *l*-ICP in serum of mice were determined by HPLC. The results showed that administration of *l*-ICP before cocaine intake once per day for 5 days reduced cocaine-induced locomotor sensitisation on days 5 and 13 after 7 days of withdrawal. Daily pre-treatment with *l*-ICP before cocaine for 6 days blocked cocaine-induced CPP, while *l*-ICP itself did not cause any preference or aversion. Such finding suggested that *l*-ICP may act as a D_1_ partial agonist and a D_2_ antagonist *in vivo*, and may be a promising agent for the treatment of cocaine addiction. Interestingly, some studies found that *l*-THP attenuates the brain stimulation rewards associated with cocaine use (Xi et al. 2007) and the self-administration of cocaine and promotes the recovery of cocaine-induced effects in rats (Mantsch et al. 2007).

Although clinical trials examining the potential utility of *l*-THP for cocaine addiction have not been conducted, which is primarily due to the lack of approval for testing in the US, the results of studies that test for its effectiveness in heroin-dependent populations have been promising.

Yang et al. (2008) used *l*-THP to treat opioid heroin withdrawal syndrome. Briefly, 120 hospitalized patients on heroin were treated orally with 60 mg of *l*-THP twice daily for one month in a recent double-blind clinical trial in China. Heroin Withdrawal Scale questionnaires (HWC) were used to measure protracted abstinence Withdrawal syndrome (PAWS), and withdrawal rate was indicated by a positive urine test. The study found that *l*-THP significantly reduced heroin craving and withdrawal symptoms and improved the abstinence rate in heroin addicts. Hu et al. (2006) examined the detoxification effect of *l*-THP combined with methadone on heroin. Sixty patients were randomly divided into the methadone plus *l*-THP treatment group and methadone group. The results showed that the combination group had a 96% success rate in detoxification, while the methadone group had a 73% success rate in detoxification. Total methadone use was also significantly reduced in the combination group.

### Effect on the circulatory system

#### Effect on the cardiovascular system

##### Anti-arrhythmic activity

Arrhythmia is an important group of diseases in cardiovascular disease. It is defined as the abnormal frequency or rhythm of heartbeat caused by a conduction defect in heart activity. Wang and Li (1987) used the ouabain-induced rabbit arrhythmia model as the research object and studied the anti-arrhythmia effect of *l*-THP. The results showed that intravenous *l*-THP (6 mg/kg) could reverse the ventricular tachycardia (VT) induced by ouabain injection in rabbits and exert an anti-arrhythmia effect. Dai et al. (1996) studied the effect of the THB extract, CPU 86017, on ion channels in arrhythmia animal models with electrophysiological approaches. The results showed that CPU 86017 prolonged the mono‐phasic action potential duration (MAPD) in rabbit heart and the action potential duration (APD) of isolated myocytes in guinea pigs. CPU 86017 blocked the delayed outward rectifier current [I_K_] by 30% ± 4% and the I_K_ tail by 16% to 59% at 3–30 μM. Therefore, CPU 86017 may be a complex class III anti-arrhythmic drug with properties of class I and class IV anti-arrhythmic drugs. Luo (2016) used rabbits as research objects to observe the changes in the ratio of diastolic and systolic phases (D/S) of rabbits after intravenous infusion of THP. The results showed that the D/S ratio at 10 and 30 min after THP injection significantly decreased, and the heart rate decreased.

#### Effect on the myocardium

Min et al. (2001) used isoprenaline (ISO) to create rat models of myocardial ischaemia necrosis injury and then applied *l*-THP to observe its protective effect on the myocardium. The results indicate that *l*-THP can significantly reduce the myocardial necrosis area, inhibit myocardial tissue creatine phosphate kinase (CK) and lactate dehydrogenase (LDH) release, lower levels of serum CK and LDH, protect myocardial tissue superoxide dismutase (SOD) activity, reduce malondialdehyde (MDA) levels, and lower levels of serum free fatty acid (FFA), thereby protecting the myocardial function.

The main apoptosis gene in the human body, Bax, belongs to the B-cell lymphoma-2 (Bcl-2) gene family. The overexpression of Bax can antagonise the protective effect of Bcl-2 and lead to cell death. Ling et al. (2006) investigated the myocardial protective activity of RC extract in rats with myocardial I/R injury under thoracotomy anaesthesia. The left anterior descending (LAD) coronary artery occlusion pre-treated with the RC extract could significantly reduce infarct size and improve heart function. Furthermore, greater level of the apoptosis protein, Bcl-2, and attenuated expression of Bax were found in rats treated with the RC extract. Therefore, the extract from RC exerted a protective effect in the myocardial I/R injury rat model by inhibiting myocardial apoptosis through the modulation of the Bcl-2 family.

The PI3K-Akt-eNOS-NO pathway has been reported to play a protective role in myocardial I/R (Gao et al. 2002). Han et al. (2012) investigated the protective effect of *l*-THP on myocardial I/R injury. They established the myocardial I/R model by blocking the LAD coronary artery for 30 min and allowing reperfusion for 6 h. *l*-THP activated the PI3K/Akt/eNOS/NO pathway and increased the expression of hypoxia inducible factor-1 (HIF-1α) and vascular endothelial growth factor (VEGF) while suppressing iNOS-derived NO production in the myocardium; reducing the accumulation of inflammatory factors, including myeloperoxidase and TNF-a; and alleviating apoptosis, ultimately promoting the protective effect of *l*-THP against myocardial I/R injury.

Additionally, RC has significant therapeutic effects on heart failure after myocardial infarction. In fact, Wu et al. (2007) found that the oral ethanol extract of RC (50, 100, or 200 mg/kg/day for 8 weeks in a rat heart failure model) significantly decreased left ventricular end diastolic pressure from 19 ± 5 mmHg to 12 ± 2 and 9 ± 3 mmHg, respectively, reduced the myocardial infarction, area lung/body weight ratio, and left ventricular (LV)/body weight ratio. Activation of neurohormones was also found to be inhibited while cardiac function was improved. They believed that RC could inhibit myocardial cell apoptosis by regulating the expression of Bcl-2 family, thereby protecting against heart failure caused by myocardial infarction in rats.

Long-term pressure overload may easily cause cardiac hypertrophy. Wen et al. (2007) established an animal model of transverse abdominal aorta constriction (TAAC) in rats. Briefly, rats were given the RC alcohol extract (200 or 50 mg/kg/day for seven weeks) from the second week after induction of pressure overload to investigate the protective effect of RC on cardiac hypertrophy. The results showed that the LV collagen volume fraction (CVF) of rats was reduced after treatment with the RC ethanol extract. This occurrence reduced the levels of type I collagen on pressure-overloaded cardiac hypertrophy induced by transverse abdominal aorta constriction in rats, reducing the degree of myocardial fibrosis, improving cardiac function, and playing a role in the prevention of myocardial hypertrophy.

#### Dilated coronary artery

He et al. (2017) reported that the RC extract can significantly expand the coronary vessels in rabbit and cat heart, reduce coronary resistance, increase coronary blood flow, and significantly improve the tolerance of mice to normobaric or hypobaric hypoxia.

#### Effect on cerebral I/R injury

Ischaemic stroke refers to hemiplegia and disturbance of consciousness caused by cerebral infarction and cerebral artery occlusion based on cerebral thrombosis. Therefore, there is a clinical need for a drug that can treat and prevent cerebral I/R injury. Experiments have shown that RC has a protective effect on cerebral I/R injury and has a good applicability in the prevention and treatment of cerebral I/R injury (Wang et al. 2003). Liu and Yang (2004) established a global cerebral I/R model in rats by using the Pulsinelli’s four-vessel occlusion method (Pulsinelli et al. 1982). The expression level of Bcl-2 and Bax mRNA was detected by in situ hybridisation and reverse transcriptional polymerase chain reaction (RT-PCR). The number of apoptotic neurons was examined by the TUNEL method. Based on the results, *l*-THP could ameliorate cerebral I/R damage by reducing apoptosis through the regulation of Bcl-2 and Bax. Yang et al. (2000) examined the effect of *l*-THP on neuronal apoptosis in acute cerebral I/R rats. They found that treatment with *l*-THP could increase the number of neuron survival and heat shock protein (HSP) 70 positive cells. Studies showed that *l*-THP could reduce apoptosis and necrosis of neurons, upregulate the expression of HSP70, and protect against ischaemic brain injury. Mao et al. (2015) established a permanent middle cerebral artery occlusion (MCAO) model in rats, and showed that I/R induced albumin leakage, Evans blue extravasation, increased cerebral water content, decreased cerebral blood flow, cerebral infarction area, and neurological impairment were reduced after *l*-THP treatment. *l*-THP also inhibited the down-regulation of tight junction (TJ) proteins, matrix metalloproteinases-2/9 (MMP-2/9), Src kinase phosphorylation, and caveolin-1 activation. The Src kinase inhibitor, PP2, could reduce Evans blue dye extravasation and inhibited the activation of MMP-9 and caveolin-1, and down-regulated the expression of occludin after I/R. In summary, *l*-THP could protect against cerebral I/R injury and reduce cerebral blood-brain barrier (BBB) injury and cerebral oedema. This might be because *l*-THP initiates this protective effect by binding to the Src kinase, inhibiting the phosphorylation of Src kinase.

#### Antihypertensive activity

Hypertension is a chronic condition where arterial blood pressure is persistently elevated. Uncontrolled high blood pressure can lead to serious damages to the heart, brain, kidneys, and other organs and lesions, such as stroke, myocardial infarction, kidney failure, etc. (Campbell et al. 2015). Chueh et al. (1995) found that the intravenous administration of *l*-THP (110 mg/kg) could cause hypotension, bradycardia, decreased release of hypothalamic serotonin and norepinephrine, and increased release of hypothalamic dopamine in rats. *l*-THP could induce hypotension and bradycardia by inhibiting 5-HT_2_ and/or D_2_-receptor in the hypothalamus of rats.

Abnormal vasoconstriction and diastolic function are some of the indicators of the pathogenesis of hypertension. Normal endothelium function plays a vital role in vascular physiological homeostasis and vascular relaxation. Zhou et al. (2019) studied the endothelial dependent and independent vasodilation of the aorta in rats and found that THP can improve the contractility of rat aorta induced by phenylephrine (Phe), KCl and U46619. The relaxation effect of THP on rat aorta was endothelium-dependent and independent. To add, the underlying mechanism of THP relaxing rat aorta involved the PI3K/Akt/eNOS/NO/cGMP signalling pathway, Ca^2+^ channels, and K^+^ channels rather than the β-adrenergic receptor, COX_2_, and the renin-angiotensin system (RAS). These findings indicated that THP might be a potent treatment for diseases with vascular dysfunction, such as hypertension.

#### Antithrombotic activity

Thrombosis refers to the formation of blood clots in blood vessels that impede the flow of blood in circulation. Activation and aggregation of platelets are key to thrombosis. Hence, Guo et al. (2000) explored the effect of DHC on cAMP mass of concentration in rabbit platelets, and found that DHC increased platelet cAMP level in rabbits and could significantly inhibit the release of ADP-induced platelet factor 4 (PF4) and the generation of thromboxane A_2_ (TXA_2_). Zhang et al. (2016) used the YHTA as raw materials and then employed platelet bio-specific extraction and HPLC-DAD/LC-MS analysis to screen five active alkaloids of antiplatelet aggregation of Corydalis, namely, glaucine, DHC, THB, THC, and corydaline. Using the *in vitro* anti-platelet aggregation activity test, the low doses of the above five alkaloids were found to inhibit platelet aggregation induced by thrombin (IC_50_ values of glaucine, DHC, THB, THC and corydaline were 49.06, 34.91, 33.55, 84.26, and 54.16 μg/mL, respectively), and play a good role in the antithrombotic effect. Li et al. (2016) reported that RC binds to the P2Y12 receptor and mediate Gαi proteins, activating AC and/or PI3K downstream signalling pathway, thus inhibiting platelet aggregation. Tan et al. (2019) further investigated the mechanism and corresponding signal cascade of two active alkaloids in RC: DHC and THB behind the inhibition of platelet aggregation. The results demonstrated that DHC could inhibit platelet aggregation by binding to the adenosine diphosphate (ADP) receptors, P2Y1 and P2Y12. To add, THB might also bind to the THR receptor PAR1 by mediating the Gi signalling pathway, thus affecting platelet function.

### Effect on the digestive system

#### Antigastrointestinal ulcer activity

*Helicobacter pylori* infection has been ascertained to be an important etiologic impetus that usually leads to chronic active gastritis and gastric ulcer. Li et al. (2005) use a *Helicobacter pylori* standard and five clinical strains as experimental pathogens, and employed the RC ethanol extract to evaluate its antibacterial activity *in vitro*. The results showed that the Corydalis alkaloids had an inhibitory effect on pyloric ligation ulcer, waterlogged stress ulcer, histamine ulcer, and acetic ulcer in rats, but no effect on reserpine ulcer. Xu et al. (2015) investigated the protective effects of RC and *l*-THP on gastrointestinal injury in morphine-dependent rats. Thus, they found that RC and *l*-THP could significantly reverse the abnormal decrease in dopamine transmitter and abnormal increase in D_2_R in the stomach and duodenum of morphine-dependent rats. Such occurrence resulted in a protective effect in the gastrointestinal tract. In addition, Li et al. (2012) reported that DHC can block the release of noradrenaline from the adrenergic nerve terminals in both the *Taenia caecum* and pulmonary artery, thereby inhibiting the relaxation or contraction of adrenergic neurons and pain relief, ultimately preventing the occurrence of experimental ulcers.

#### Effect on liver

Min et al. (2006) explored the effects of *l*-THP on liver injury induced by carbon tetrachloride (CCl_4_) in mice. Briefly, mice were administered *l*-THP i.p. 20 and 40 mg/kg daily for 9 d. Thereafter, an acute liver injury model was established with 0.1% CCl_4_ i.p. 20 mL/kg. *l*-THP significantly reduced the level of serum alanine aminotransferase (ALT) and aspartate aminotransferase (AST) after 17 h, inhibited lipid peroxidation in the liver, and increased SOD activity in the liver tissue of the model. Evidently, degeneration of hepatocytes was prevented in mice treated with *l*-THP, and the liver histological structure was well maintained.

### Antimicrobial, anti-inflammation, and antiviral activity of RC

The RC extract has strong antibacterial activity. In particular, Ber has significant inhibitory effect on dysentery bacillus, pneumococcus, typhoid bacillus, and other bacteria (Li et al. 2016). *Clostridium perfringens* is a common clinical Clostridia that produces a large amount of gas, leading to severe emphysema in tissues, which in turn affects blood supply and causes extensive necrosis in the tissues. The neuraminidase (NA) protein of *C. perfringens* plays an important role in bacterial proliferation and is considered a novel antibacterial drug target. Kim et al. (2014) isolated four isoquinoline alkaloids from RC, namely pseudocoptisine, glaucine, corydaline, and THP and found that they could inhibit the activity of NA and the proliferation of the bacteria.

*Helicobacter pylori* infection is one of the important causes of chronic active gastritis and gastric ulcer. Li et al. (2005) evaluated the antibacterial activity of RC ethanol extracts in vitro by using *Helicobacter pylori* as the standard and 5 clinical strains as experimental pathogens. As a result, the ethanol extract of RC was found to show mild antibacterial activity to all tested strains, with a minimum inhibitory concentration (MIC) of 60 µg/mL.

Inflammation is an adaptive tissue response triggered by harmful stimuli and conditions, such as tissue damage or microbial infection. Studies have shown that the RC methanol extract and its alkaloid components, dehydrofumarine, d-glaucine, and *l*-THB can be considered to exert anti-inflammatory activity (Kubo et al. 1994). Ishiguro et al. (2011) found that DHC inhibited the elevation of mitochondrial membrane potential and adenosine triphosphate (ATP) depletion in macrophages stimulated by lipopolysaccharide (LPS). However, neither affected basal mitochondrial membrane potential nor ATP content in non-stimulated macrophages. DHC also inhibited the increased concentrations of the pro-inflammatory cytokines, interleukin (IL)−1β and IL-6, in the culture media of LPS-stimulated macrophages, thereby producing an anti-inflammatory effect. Oh et al. (2010) detected THP’s dose-dependent inhibitory effect on the LPS-induced pro-inflammatory cytokine, IL-8, by ELISA, reverse transcription-polymerase chain reaction, mitogen-activated protein kinase (MAPK) activation, and WB analysis. The results showed that pretreating THP-1 cells with 0.2, 1, or 2 mM of THP significantly inhibited IL-8 secretion induced by LPS. THP also exerted an anti-inflammatory effect by blocking MAPK phosphorylation in the human monocytic cell line, THP-1. By using the mouse auricular swelling model which was established with xylene, Qiu et al. (2009) administered an intravenous injection to Ivans rat via the caudal vein and revealed the effects of RC processed with vinegar and cleansing RC to ooze-induced by inflammation. The results showed that both RC processed with vinegar and cleansing RC had obvious inhibitory effects on mouse auricular swelling induced by dimethyl benzene and could inhibit ooze in Ivans rats. Compared to the cleansing RC group, the difference in anti-inflammatory action was not significant (*p* > 0.05). Based on NF-κ B suppression, Yang et al. (2015) found that *l*-THP could be a promising compound in the prevention and treatment of early vascular inflammatory reaction in atherosclerosis by inhibiting monocyte adhesion to vascular endothelial cells through the downregulation of ICAM_-1_ and VCAM_-1_ in vascular endothelial cell.

Wang and Ng (2001) demonstrated that alkaloids from RC exerted some inhibitory actions on human immunodeficiency virus type 1 (HIV-1) reverse transcriptase. In fact, at a concentration of 5 mg/mL, it could inhibit HIV-1 reverse transcriptase by approximately 50%.

### Anticancer activity

Recently, RC was found to have certain therapeutic effects on many types of tumours. Corydalis alkaloids, such as corydalis, THP, Ber, DHC, and 13-methyl-palmatrubine, display certain anti-tumour effects (Zhao et al. 2014; Chen et al. 2016). Zhao (2015) found that THP could inhibit the proliferation of U251MG malignant glioma cells *in vitro* and promote their apoptosis. *In vivo*, the growth of malignant glioma tissue was obviously inhibited, and the survival time of tumour-bearing mice was extended.

Ber, an alkaloid in RC, induced the apoptosis of human cancer cells, such as the human colon cancer cell, HCT116, nasopharyngeal carcinoma cell, HONE1, hepatoma carcinoma cell, HepG2, and colon cancer cell, SW480 (Lin et al. 2004; He et al. 2005; Hwang et al. 2006; Letasiova et al. 2006; Tsang et al. 2009). Ber also inhibited cell invasion in non-small lung cancer (Peng et al. 2006).

Lung cancer is the second largest cancer in the world, with an incidence rate that is increasing year by year in developing countries such as China. Chen et al. (2016) found that 13-methyl-palmatrubine isolated from RC could dose-dependently inhibit apoptosis and cell cycle arrest in lung cancer A549 cells. To add, 13-methyl-palmatrubine inhibited the growth of A549 cells, which was mediated by the blocking of the epidermal growth factor receptor (EGFR) signalling pathway and activation of the MAPK signalling pathway.

RC exerts strong anticancer activities against human breast cancer MDA-MB-231 cells. Zhao et al. (2014) examined the synergistic cytotoxicity effect of three natural compounds, DHC, Ber, and THP, isolated from RC. Their results revealed that the combination of Ber and THP had the strongest anti-cancer cell proliferation effect at a ratio of 2:3 (Ber:THF, average CDI value = 0.5795). Xu et al. (2012) found that DHC isolated from RC could induce apoptosis of the human breast cancer cell line, MCF-7, ultimately inhibiting their proliferation.

*l*-THP has a certain inhibitory effect on abdominal aortic aneurysm (AA). Hence, Wang et al. (2018) evaluated the effects of *l*-THP on AA progression in experimental rats induced with perfusion of elastase. After treatment with *l*-THP, aortic diameter (ADs) significantly reduced, systolic blood pressure (SBP) reduced, and the expression of metalloproteinase and monocyte chemotactic protein-1 were decreased. To add, the tissue samples were found to have a significant reduction in the levels of iNOS compared to the control group. Therefore, *l*-THP inhibits the progression of AAs by suppressing MMP and monocyte chemotactic protein-1 in rats.

Tumour multi-drug resistance (MDR) has become an anti-infection drug therapy and the main impediment to tumour chemotherapy failure. Therefore, the search for new scaffolds with natural or synthetic compounds is a hotspot in MDR research. The major mechanism leading to cancer cell MDR is the overexpression of P-glycoprotein (P-gp) and MDR-associate protein 1 (MRP1). These transporters reject anti-cancer drugs and greatly affect the efficacy of chemotherapy (Gottesman 2002). Zhang et al. (2005) found that *l*-THP, an active component extracted from RC, has anti-MDR effects in the MCF-7 cell line. Besides, it could interact with P-gp and alter its ATPase activity to reverse MDR. *l*-THP was also identified to enhance vincristine’s ability to inhibit the proliferation of human leukaemia cell lines. Lei et al. (2013) extracted the active component of RC, glaucine, and determined its reversal properties by using the drug-resistant cancer cell lines, MCF-7/ADR, and the corresponding parental sensitive cells. The results showed that glaucine could competitively inhibit two transporters involved in MDR, namely P-gp and MRP1-mediated efflux and activate the ATPase activities of the transporters. These findings indicate that glaucine is a substrate that competitively inhibits P-gp and MRP1, thereby reducing the MDR of cancer cells and improving the efficacy of chemotherapy on cancer cells. In the MCF-7/ADR cancer cell lines, the IC_50_ values of Adriamycin and mitoxantrone respectively decreased from 41 and 24.19 µg/mL to 12.6 and 2.11 µg/mL, with 12.5 µM didehydropapaverine. Glaucine was also found to suppress the expression of ATP-binding cassette (ABC) transporter genes and effectively reverse the resistance of MCF-7/ADR to Adriamycin and mitoxantrone.

In addition, a recent study demonstrated that alkaloid extract of RC could significantly inhibited VEGF-induced signalling pathways and attenuated the levels of downstream regulators phospho-ERK1/2, phospho-STAT3, and phospho-AKT in human umbilical vascular endothelial cells (HUVECs), thereby inhibiting angiogenesis, provides potential for inhibiting tumour angiogenesis (Wan et al. 2019).

### Other activities

Yu et al. (2014) found that the alcohol extract of RC could significantly reduce the blood glucose level of normal and diabetic mice and induce glucose tolerance in insulin-resistant mice with high fat. Hence, this extract was identified to have potential therapeutic effects on diabetes and its complications. Wangchuk, Keller, Pyne, Willis, et al. (2012) found that protopine, the isoquinoline alkaloids isolated from *Corydalis dubia*, had antimalaria effects. Yu et al. (2010) also found that THP has protective effects against γ-ray-induced damage to human endothelial cells.

## Conclusions and future perspectives

The resources available to RC are abundant. In the TCM classification, it belongs to the blood circulation drugs. In traditional medicine, RC is mostly vinegar processed decoction pieces, which can promote blood circulation, remove blood stasis, and relieve pain. Modern pharmacological studies have found that the alkaloid components of RC have a variety of biological activities, primarily focussing on the study of cardiovascular, cerebrovascular and nervous systems, echoing traditional medicine. Its main active ingredient is alkaloids, with total alkaloids accounting for more than 2% of its content. Over 80 types of alkaloids have been isolated and identified. Indeed, RC exhibits strong pharmacological effects and minimal side effects (Wang et al. 2016). In the past 30 years, researchers have performed many experimental investigations to prove the pharmacological effects of *Corydalis analgesia*, as well as its sedative and anti-inflammatory effects (CPC 2015). In recent years, with the advancement of science and technology, pharmacological research on the effective parts and certain monomer components of RC have been performed, and its mechanism of action has been further clarified from the perspective of molecular biology.

Although RC and its alkaloids have been used to treat several diseases and have been extensively studied in animal studies, these current findings still have some limitations. The current research focussed on alkaloids, such as *l*-THP, THB, and DHC, whereas studies on the pharmacological activities of the YHTA and other compounds are relatively rare and progress slowly. This study provided challenges for the study of RC, and opportunities for drug development and clinical application in the future.

In summary, current pharmacological studies on RC mainly focus on the nervous system, circulatory system, digestive system, other endocrine systems, its anti-cancer effects, and drug withdrawal. Besides, the mechanism of action of its effective part is mainly focussed on YHTA, D-corydaline, THP, *l*-THP, coptisine, glaucine, palmatine, and other ingredients. The pharmacological action of RC is multi-targeted while the pharmacological effects of each component are additive. Although experimental data support the beneficial effects of these drugs in the treatment of myocardial ischaemia, ischaemic stroke, thrombosis, cancer, neurodegenerative diseases, depression, and other diseases, its physiological activity remains a concern. In this article, we reviewed the ongoing progress of the phytochemical and the pharmacological effects of RC and aimed to lay the foundational guidelines for the clinical application of RC as well as further research and development of new drugs stemming from RC.
